# Genomic analysis of Latin American-Mediterranean family of *Mycobacterium tuberculosis* clinical strains from Kazakhstan

**DOI:** 10.1590/0074-02760200215

**Published:** 2020-09-18

**Authors:** Pavel Tarlykov, Sabina Atavliyeva, Arike Alenova, Yerlan Ramankulov

**Affiliations:** 1 National Center for Biotechnology, Nur-Sultan, Kazakhstan; 2 National Scientific Center for Phthisiopulmonology, Almaty, Kazakhstan

**Keywords:** Mycobacterium tuberculosis, genome, tuberculosis, phylogeny

## Abstract

The human-adapted strains of the *Mycobacterium tuberculosis* complex (MTBC) comprise seven phylogenetic lineages originally associated with their geographical distribution. Here, we report the genomes of three drug-resistant clinical isolates of the Latin American-Mediterranean (LAM) family collected in Kazakhstan. We utilised whole-genome sequencing to study the distribution and drug resistance of these isolates. Phylogenetic analysis grouped the genomes described in this study with the sequences from Russia, Uzbekistan, and Kazakhstan belonging to the LAM family. One isolate has acquired extensive drug resistance to seven antituberculosis drugs. Our results suggest at least two multi-drug resistant (MDR)/extensively drug-resistant (XDR)-associated genotypes of the LAM family circulate in Kazakhstan.

*Mycobacterium tuberculosis* is a human pathogen with diverging lineages initially associated with a specific geographic region.^1^ The lineages present in the human-adapted *M. tuberculosis* complex (MTBC) are distinguished as ancient and modern. The ancestral lineages include lineage 1 (Indo-Oceanic), lineage 5 (West Africa 1), lineage 6 (West Africa 2), while recently discovered lineage 7 (Ethiopia) appears to be intermediate between the ancient and modern ones.^2^ Modern lineages include lineage 2 (East-Asian), lineage 3 (East-African-Indian), and lineage 4 (Euro-American), which includes the Latin American-Mediterranean (LAM) family. The LAM family was first discovered based on the strain collection whose descent was mainly Latin America and the Mediterranean area.^3^ ^,^ ^4^ Human migrations have led to a recent expansion of the LAM family worldwide. In 2014, the LAM family has already been observed in 47 countries with different prevalence rates, including several Central Asian countries (Kazakhstan, Uzbekistan, and Turkmenistan) and neighboring Russia.^5^ To date, LAM is the most predominant family of *M. tuberculosis* observed in Kazakhstan after the Beijing family.^6^ Region of difference (RD) loci divided the LAM lineage into several sublineages, namely RD-Rio, RD174, and RD115. RD-Rio sublineage is defined by 26 kb deletion and generally concomitant with large deletion RD174.^7^ The other sublineage is characterised by deletion RD115, which includes the LAM-RUS branch with a specific insertion of IS*6110* into the *plcA* gene.^8^

Kazakhstan is among the 30 countries with the highest burden of multidrug-resistant tuberculosis in the world.^9^ A recent expansion of drug-resistant isolates in the country is linked to the spread of “successful” Central Asian/Russian sublineage of *M. tuberculosis* Beijing genotype.^10^ At the same time, a number of studies have reported rapid acquisition of drug-resistance in the LAM family including KZN isolates from South Africa and LAM-RUS sublineage widespread in Kazakhstan and its neighboring countries.^8^ ^,^ ^11^ In previous studies, the LAM genetic family was found at a proportion of 11% in a sample of 470 *M. tuberculosis* isolates from 12 provinces of Kazakhstan (51/470 = 10.85%).^6^ ^,^ ^12^ The other study describes local LAM isolates represented mainly by RD115 LAM-RUS sublineage (29/30 = 96.67%).^13^ Additionally, recent findings observed a very first isolate with an RD-Rio deletion (LAM RD-Rio) not endemic to Central Asia.^14^ Nevertheless, genomic data on the LAM isolates circulating in Kazakhstan is very limited. Very few local collections of MTBC isolates were tested for RD loci or IS*6110* insertions.^13^ Furthermore, there are only three whole-genome sequences (WGS) of the LAM isolates from Kazakhstan published to date.^5^ The additional genomes will provide more data on the genetic variations occurring in drug-resistant LAM family isolates circulating in Kazakhstan and neighboring countries.

Thus, we provide the whole-genome sequencing data of three LAM isolates from a collection of drug-resistant *M. tuberculosis* isolates collected in Nur-Sultan city, Kazakhstan. Epidemiology of *M. tuberculosis* including lineage 4 is routinely studied by the analysis of single nucleotide polymorphisms (SNP), RD loci, IS*6110*-restriction fragment length polymorphisms (RFLP), spoligotyping, and/or mycobacterial interspersed repetitive units (MIRU)-typing.^15^ Some of these conventional molecular genotyping methods are laborious and have various limitations.^16^ In this study, we have implemented whole genome sequencing with *in silico* analysis of the epidemiological characteristics of the local LAM isolates.

A collection of 28 drug-resistant *M. tuberculosis* strains was isolated in Nur-Sultan, Kazakhstan, from sputum of patients with clinically suspected tuberculosis. The drug susceptibility was tested using a Bactec MGIT 960 culture system (Becton, Dickinson) according to the manufacturer’s protocol. DNA was extracted using the cetyltrimethylammonium (CTAB) procedure.^17^ The quality of the DNA was checked using a Qubit double-stranded DNA (dsDNA) high-sensitivity (HS) assay kit (Thermo, Massachusetts, USA) and a Qubit 2.0 fluorometer (Thermo). A SNP real-time polymerase chain reaction (PCR) assay for detection of G to A transition in *fbpC* codon 103 (*Rv0129c*) was carried out using the CFX96 Touch System (Bio-Rad, California, USA). Three LAM isolates were SNP-confirmed for sequencing (no. 3538, 4142, and 4330).

Isolates no. 3538 and 4142 were sequenced using a MiSeq platform (Illumina, California, USA) and the other LAM isolate no. 4330 using an Ion Torrent platform (Thermo). For the MiSeq sequencing, libraries with an average fragment size of 600 bp were prepared using a Nextera DNA Flex Library Prep kit (Illumina) according to the manufacturer’s instructions. A barcoded library for the isolate no. 4330 was prepared using an Ion Xpress Plus fragment library kit and an Ion Xpress barcode adapters 1-16 kit (Thermo). The median library size of 480 bp for the adapter-ligated 400-base-read library was size-selected with E-Gel SizeSelect II Agarose Gel (Invitrogen, California, USA). The sequencing was conducted on the Ion Torrent PGM sequencing platform using a Hi-Q sequencing kit (Thermo) and a 318 Chip (Thermo) as described previously^18^ The quality of the raw sequencing data was checked using FastQC v.0.11.9.^19^ Raw sequence reads filtered with Trimmomatic v.0.38 (Phred score > 20) were used for further nucleotide variation analysis and *de novo* assembly with SPAdes v.3.14.1.^20^ ^,^ ^21^ PhyResSe online tool was used to check for heteroresistance in the obtained WGS data.^22^ The annotation of genomes was performed by either NCBI Prokaryotic Genome Annotation Pipeline (paired-end reads) or Prokka annotation pipeline v.1.14.5 (single-end reads).^23^ ^,^ ^24^ Default parameters were used for all software unless otherwise specified.

An *in silico* spoligotyping was performed by SpoTyping 2.1 and the assignment of regions of deletion was accomplished by TB-Profiler.^25^ ^,^ ^26^ The reads also were mapped to the reference genome H37Rv (Genbank accession no. NC_000962.3) to confirm IS*6110* insertions, and spoligoprofiles with a set of primers by Kamerbeek, J et al.^27^ using Geneious Prime v.2019.2.1.

An SNP matrix was produced by comparing SNPs found between the studied genomes, and sequences of 81 MTBC isolates [Supplementary data (Table)]. The filtered sequence reads were mapped using the BWA-MEM program with *M. tuberculosis* H37Rv (NC_000962.3) reference genome sequence.^28^ SNPs were called by UnifiedGenotyper pipeline (GATK v.3.8.1.0).^29^ SNPs located in repetitive genome regions, PE/PPE genes of the reference genome (NC_000962.3) were filtered by TB Variant Filter v.0.1.3 before compiling the concatenated sequence.^30^ ^,^ ^31^ Variant calls with per-base coverage of fewer than 10x coverage depth or a Phred score below 20 were removed. The obtained high-confidence SNPs were subsequently written to a multi-FASTA alignment. A maximum-likelihood tree of all concatenated SNPs was generated using RAxML v8.2.11 with 100 bootstrap iterations.^32^ We used the General Time Reversible (GTR) model of nucleotide substitution implemented in RAxML. The phylogeny was rooted using *Mycobacterium canettii* as an out-group. The phylogenetic tree was visualised with FigTree software v.1.4.4.

Characteristics of the draft whole-genome sequences of the LAM isolates are listed in Table I. The whole-genome shotgun sequencing data gave an average 110-fold genome coverage. MiSeq instrument generates sequence reads from both ends of a fragment (paired-end reading); while Ion Torrent produces single-end reads. Sequencing from both ends of fragment produce reads capable of accurate detection of genomic rearrangements and repetitive sequence elements.^33^ The library preparation procedure took two days for both platforms, while MiSeq sequencing took a longer time to perform than single-end Ion Torrent technology (38 h versus 8 h). The lowest coverage (~ 84x) was obtained for single-end sequenced DNA from isolate no. 4330 that have resulted in a higher number of the assembled contigs, as shown in Table I.

**TABLE I:** Characteristics of three *Mycobacterium tuberculosis* genome assemblies

Isolate	Platform	SRA accession No.	Genome size (bp)	GC content (%)	Coverage (x)	No. of contigs	Total no. of CDSs	SNPs called*
3538	MiSeq**	SRR11241401	4,427,028	65.24	130	404	4,392	1455
4142	MiSeq	SRR11241400	4,351,634	65.58	116	243	4,237	1185
4330	Ion Torrent***	SRR11241402	4,344,962	65.00	84	925	4,495	989

*: compared to the genome of reference strain H37Rv (GenBank accession no. NC_000962.3); **: MiSeq libraries were prepared using a Nextera DNA Flex Library Prep Kit and the sequencing was performed using MiSeq Reagent Kit v3; ***: Ion Torrent library was prepared using an Ion Xpress Plus fragment library kit and the sequencing was conducted using a 318 Chip. CDSs: coding sequences; GC: guanine-cytosine; SRA: sequence read archive.

Three isolates harbored high-confidence mutations in various genes associated with the drug resistance compared to the genome of reference strain H37Rv (GenBank accession no. NC_000962.3) (Table II).^34^ As follows from Table II, the studied isolates were not having a mixture of wild-type and mutant alleles also known as heteroresistance. No mixed calls were assigned in the alleles associated with the drug resistance with minority alleles composed more than 5% of the read depth. The isolates’ phenotypic susceptibility was tested for isoniazid (INH), rifampin (RIF), streptomycin (SM), ethambutol (EMB), amikacin (AMI), kanamycin (KAN), and ofloxacin (OFX). WGS has confirmed phenotyping results for multi-drug resistant (MDR) isolates no. 3538 and 4330 and extensively drug-resistant (XDR) isolate no. 4142. Genotypic prediction of the *M. tuberculosis* susceptibility to anti-TB agents was found to correlate with phenotypic susceptibility.^35^ It confirms predictive WGS-based drug-resistance profiling as a valuable tool for clinical use.

**TABLE II:** Mutations observed in drug resistance-associated loci of the *Mycobacterium tuberculosis* isolates

Isolate	Drug resistance							
	AMI	EMB	INH	OFX	PZA	RIF	SM	ETH
3538								
Gene			*katG*			*rpoB*		
AA change			Ser315Thr			Ser450Leu		
Base change			G/C 944			C/T 1349		
% of reads			100			100		
4142								
Gene	*rrs*	*embB*	*katG*	*gyrA*		*rpoB*	*rrs*	*fabG*
AA change	-	Met306Ile	Ser315Thr	Asp94Tyr		His445Leu	-	-
Base change	A/G 1401	G/C 918	G/C 944	G/T 280		A/T 1334	A/C 514	C/T -15
% of reads	100	100	100	100		99	96	100
4330								
Gene		*embB*	*katG*			*rpoB*	*rrs*	*fabG*
AA change		Met306Ile	Ser315Thr			His445Leu	-	-
Base change		G/C 918	G/C 944			A/T 1334	A/C 514	C/T -15
% of reads		98	99			100	99	100

AMI: amikacin; EMB: ethambutol; INH: isoniazid; OFX: ofloxacin; PZA: pyrazinamide; RIF: rifampin; SM: streptomycin; ETH: ethambutol.

The prevalence rate of the LAM isolates in the current study corresponds to previously reported data (3/28 = 10.71%). The deletion of a large genomic region RD115 was observed in the three studied isolates (no. 3538, 4142, and 4330). Two out of three isolates were assigned to the LAM-RUS branch based on IS*6110* insertion in the *plcA* gene (no. 4142 and 4330). Prevalence of the LAM-RUS isolates in local samples is especially intriguing since Lineage 4 is the most heterogeneous lineage of *M. tuberculosis*, consisting of 10 different sublineages, determined by the absence of specific RD loci called RD115, RD122, RD174, RD182, RD183, RD193, RD219, RD724, RD726, and RD761.^36^ All three WGS genomes of the local LAM isolates sequenced by Stucki et al.^5^ were also defined by the large deletion RD115 (Table III). Two of them were LAM-RUS isolates with a specific insertion of IS*6110* into the *plcA* gene (G04493 and G04546).^8^ Noteworthy, RD115 was present in all LAM-RUS isolates (Table III), while two isolates with RD115 did not belong to the LAM-RUS family. Geographic mapping of the LAM-RUS family shows its prevalence across Northern Eurasia, including Russia and Kazakhstan. It was also reported in other countries, such as Brazil, Venezuela, Ethiopia, and Sierra Leone.^37^

**TABLE III:** Latin American-Mediterranean(LAM) sublineages and spoligotype international types (SITs) in *Mycobacterium tuberculosis* isolates from Kazakhstan based on the whole-genome sequences (WGS) data

Isolate	Year	Sublineage*	RD	SIT	IS*6110* (*plcA* gene) LAM-RUS	Resistance	Reference
3538	2014	L4.3.3	RD115	42	-	MDR	This study
4142	2014	L4.3.3	RD115	42	+	XDR	This study
4330	2014	L4.3.3	RD115	42	+	MDR	This study
G04485	2002	L4.3.3	RD115	42	-	MDR	Stucki et al.
G04546	2007	L4.3.3	RD115	254	+	S	Stucki et al.
G04493	2011	L4.3.3	RD115	444	+	S	Stucki et al.

*: sublineage classification based on Coll et al.^(2)^; MDR: multi-drug resistant isolate; XDR: extensively drug-resistant isolate; S: sensitive isolate.

In the current study, an *in silico* spoligotyping has classified all three isolates as a spoligotype international type (SIT) 42. SIT42 is a major ancestral spoligotype of the LAM family also known as LAM prototype spoligoprofile.^37^ These recent findings by Mokrousov et al.^37^ indicate that SIT42 was highly dominated by the LAM-RUS in the collection of *M. tuberculosis* isolates from Russia, Belarus, and Kazakhstan. Additionally, we conducted *in silico* spoligotyping for the three local LAM isolates sequenced by Stucki et al.^5^ Our *in silico* analysis suggests at least four populations of the LAM family sublineages, namely, SIT42, SIT42/LAM-RUS, SIT254/LAM-RUS, and SIT444/LAM-RUS, circulate in Kazakhstan based on the WGS data (Table III). Of these, two genotypes (SIT42 and SIT42/LAM-RUS) are associated with MDR/XDR phenotype.

In addition to spoligotyping, the presence of specific SNPs is another characteristic of the LAM isolates.^2^ ^,^ ^38^ Three studied isolates harbored the same set of LAM family-specific SNPs including G/A transition in *fbpC* codon 103 (*Rv0129c*), G/A substitution in position 8,040 (*Rv0006*), C/T substitution in position 403,364 (*Rv0338c*), G/A substitution in position 2,518,919 (*Rv2245*) and C/G substitution in position 3,426,795 (*Rv3062*). As a result, lineage and sublineage of three studied isolates were assigned as 4.3.3 based on *in silico* spoligotyping results together with phylogenetic SNPs analysis.

Finally, we conducted a maximum-likelihood phylogenetic analysis. A phylogenetic tree was constructed based on overall SNPs extracted from 84 MTBC genomic DNA sequences including WGS data from this study [Figure, Supplementary data (Table)]. This data set included fifty published genomic sequences representing phylogeny of *M. tuberculosis* lineage 4 according to Coll et al.^2^ classification. As a result, the LAM sublineage 4.3.3 was divided into several branches. The sequences no. 4142 and 4330 have formed a separate 4.3.3 LAM-RUS branch together with the published genomic sequences of isolates G04616 (Uzbekistan), G04502, and CTRI-2 (Russia), while no. 3538 has grouped with isolate G04485 (Kazakhstan). The phylogeny of the local LAM-RUS isolates fits an earlier hypothesis on the founding bacterial/human population in Northern Eurasia that was disseminated by large-scale human migration in the former Soviet Union.^37^

**Figure:**
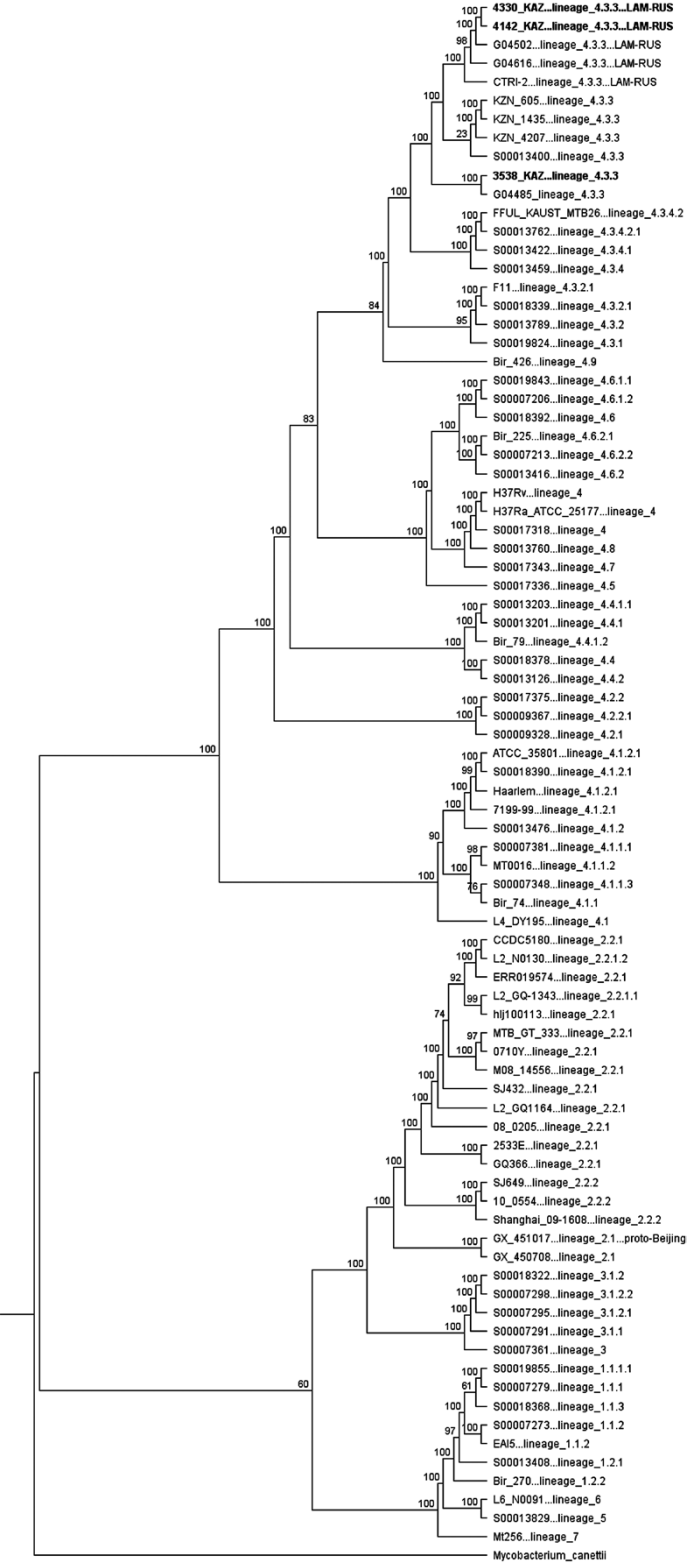
Maximum-likelihood phylogeny of three *Mycobacterium tuberculosis* isolates from this study and 81 representative genomes of *M. tuberculosis* complex (MTBC) strains. Sublineages are labeled according to Coll et al.^2^ Branch lengths are proportional to nucleotide substitutions and the topology is rooted with *Mycobacterium canettii*. Three isolates from this study are highlighted. Bootstrap values are shown.

Contribution of the present data when comparing to earlier studies is WGS-based genotypic prediction of drug-resistance combined with epidemiological genotyping that covered *in silico* spoligotyping, assignment of RD loci as well as IS*6110* insertions. The obtained genomes have provided new data on the genetic variations occurring in the drug-resistant LAM family isolates circulating in Central Asia. We found two MDR/XDR-associated genotypes of the LAM family circulating in Kazakhstan (SIT42 and SIT42/LAM-RUS). We are deeply concerned with the acquired extensive and multi-drug resistance in the local LAM family isolates. Further expansion of these drug-resistant clones represents a threat to regional TB control and the effectiveness of standardised treatment strategy. One of the limitations of the current research was the collection of genomic sequences that is not comprehensive to confirm the origin and migration pathways of the studied LAM isolates. A further comparison of the obtained WGS data with the other circulating LAM sublineage 4.3.3 isolates is needed to reveal the genetic differences responsible for the pathogenicity and transmission success of the LAM isolates.

*Accession numbers* - This Whole Genome Shotgun project has been deposited at DDBJ/ENA/GenBank under the accession no. JABACH000000000, JABACG000000000, and JABACF000000000. The raw Whole Genome Shotgun data was submitted to the NCBI SRA. SRA accession numbers are listed in Table I.
